# The growth hormone receptor gene deleted for exon three (*GHRd3*) polymorphism is associated with birth and placental weight

**DOI:** 10.1111/j.1365-2265.2011.04207.x

**Published:** 2012-02

**Authors:** Raja Padidela, Sinead M Bryan, Sayeda Abu-Amero, Rebecca E Hudson-Davies, John C Achermann, Gudrun E Moore, Peter C Hindmarsh

**Affiliations:** * Developmental Endocrinology Research Group, UCL Institute of Child Health, University College LondonLondon, UK; † Clinical & Molecular Genetics Unit, UCL Institute of Child Health, University College LondonLondon, UK

## Abstract

**Context:**

Human growth hormone receptor (GHR) transcripts have two isoforms, full-length (*GHRfl*) or exon 3 deleted (*GHRd3*). An association of these isoforms has been found with small for gestational age (SGA) infants but does not influence adult height. The role of this polymorphism in the birth size spectrum in the general population is unclear.

**Objective:**

To determine the association of maternal and infants *GHR exon 3* polymorphism with antenatal growth, birth size and early postnatal growth in two large, normal white European birth cohorts.

**Study design:**

Pregnant women from white European families were recruited by the University College London Foetal Growth Study (*n* = 774) and the Moore normal pregnancy cohort (*n* = 274). *GHR* variants, wild-type (*fl*) and deleted for exon 3 (*d3*) were analysed using multiplex PCR.

**Results:**

There was a significant underrepresentation of infants wild-type *fl/fl* (36%) and overrepresentation of *d3/d3* (14%) genotypes in the SGA infants within the cohorts (χ^2^ = 11·2, *P* = 0·003, df = 2). *Fl/fl* was overrepresented in large for gestational age (LGA) infants (χ^2^ = 6·1, *P* = 0·047, df = 2). There was a significant association of infants *GHR* isoforms with placental weight (*P* < 0·001) and birth weight standard deviation scores (*P* = 0·04) with the *fl/fl* genotype associated with a larger placental and birth weight. In multiple regression analysis, the *GHR* isoform type, maternal booking weight and parity influenced placental weight (*R*^2^ = 0·35; *P* < 0·001, df = 7). The *GHR* isoform type was not related to antenatal anthropometric measurements or growth in infancy.

**Conclusion:**

These data suggest that the *GHR* isoforms are associated with placental and birth weight.

## Introduction

The growth hormone receptor (GHR) gene (MIM: *600946) is located on chromosome 5 (5p13.1-p12) and consists of nine coding exons.^1^ In humans, *GHR* transcripts exist in two major isoforms; retention (*GHRfl*) or exclusion of exon 3 (*GHRd3*). *GHR exon 3* skipping results from a homologous recombination of two retroviral sequences flanking this exon that mimic an alternative splicing event.^1^ Historically exon 3 deletion was first reported as a potential pathological finding in two patients with GH insensitivity.^2^ Subsequent studies confirmed *GHR* exon 3 insertion/deletion as common polymorphism with these isoforms specific to individuals^3^ inherited as a Mendelian trait.^4^

During infancy and childhood pituitary, growth hormone (hGH-N) and insulin-like growth 1 (IGF-1) influence growth by virtue of hGH-N binding to the GHR. During pregnancy, hGH-N expression in the mother is suppressed; and placental growth hormone (hGH-V), a GH variant expressed by the placenta, becomes the predominant GH in the mother.^5^ hGH-V influences placental and foetal growth by binding to the maternal and placental GHR. Inherent variation in the *GHR* with insertion/deletion of an exon could explain some of the variability seen in the antenatal and postnatal growth and response to recombinant human GH (r-hGH) treatment in children with growth disorders.

Dos Santos *et al.*^6^ first reported a positive influence of *GHR exon 3* polymorphism on response to treatment with human GH in children with idiopathic short stature (ISS) and those born small for gestational age (SGA). However, no difference in the frequency of this polymorphism was found in short children compared to control adults of normal height, suggesting that this polymorphism was not primarily related to the aetiology of adult height.^6^ This lack of overall effect was postulated to be secondary to compensation by endogenous pituitary GH secretion, masking any effect of the *GHR* polymorphism on growth rate. Subsequently, a number of studies of the polymorphism frequencies in various populations of children with growth disorders have yielded inconsistent results^7^ mainly due to small sample sizes.^8^ A meta-analysis of all these studies indicates that *GHRd3* stimulates growth velocity by an additional effect of 0·5 cm during the first year of treatment with r-hGH.^7^

At present, little is known about the role of *GHR exon 3* polymorphism on antenatal and early postnatal growth with small sample sizes limiting interpretation.^9^–^11^ We have evaluated the role of *GHR exon 3* isoform on antenatal growth, birth size, placental weight and postnatal growth up to 3 years of age in two large prospectively recruited white European cohorts.

## Subjects and methods

### Study cohort

Subjects for this study were recruited from two separate white European cohorts, the University College London Hospital Fetal Growth Study (UCL-FGS) (*n* = 1650) and the Moore cohort (*n* = 310). Ethical approval was obtained from the University College London Hospitals and Queen Charlotte Hospital for Women and Hammersmith Hospital Ethics Committees. Written informed consent was obtained from all the participants on entrance into the study and then again for the participation of the infant in the study after birth. Suitable DNA samples for this study were available in 1048 infants, 774 from UCL-FGS and 274 from Moore cohort. Inclusion criteria in both cohorts were white European families presenting for the first prenatal visit before 20 weeks of pregnancy and ultrasound examination demonstrating a structurally normal single foetus. Pregnancies with antenatal complications and adverse foetal findings were excluded from the study.

From UCL-FGS antenatal ultrasonographic growth data, anthropometric measurement at birth and growth data until 3 years of age (*n* = 524) was used for the study. Data collection was standardized, and all data were collected prospectively. Antenatal growth measurements were performed by ultrasonography by a single skilled physician with expertise in foetal medicine. At birth, trimmed placental weight (stripped of blood clots, umbilical cord and membranes) was recorded. Anthropometric measures were undertaken at birth by a single observer. Weight was measured using electronic self-calibrating scales (Seca, Birmingham, UK), length by Infantometer (Child Growth Foundation, London, UK) and head circumference using a flexible metal tape. Three separate measurements were taken and the mean recorded. All measurements at birth were corrected for gestational age and sex by converting them into standard deviation scores (SDS).^12^ Similar postnatal anthropometric data were collected at six monthly intervals by a single observer up to 3 years of age.

From Moore cohort anthropometric measurements at birth were used. All families were of white European origin. Placental sample was dissected and frozen at −80 °C until use for foetal DNA preparation. Blood samples were collected in EDTA tubes from each parent for DNA extraction. Maternal weight, height, age, parity, baby’s gender, birth weight, head circumference, placental weight, past and present medical history, mode and indication delivery for delivery, pregnancy complications, smoking, diet and alcohol consumption, and partner’s medical history were collected from the mother’s notes from all families participating in the study. All measurements at birth were corrected for gestational age and sex by converting them into standard deviation scores (SDS).^12^

### Analysis of GH receptor isoforms

In the UCL-FGS cohort, genomic DNA was extracted from cord blood (*n* = 449) and from placenta (*n* = 325) where suitable cord blood sample was not available. In the Moore cohort, genomic DNA samples were obtained from placental tissue (*n* = 274). Placental samples were dissected near the umbilical cord insertion point, snapped frozen in liquid nitrogen and stored at −80 °C until use for foetal DNA preparation. DNA was extracted from the maternal blood samples and stored for analyses of maternal genotype.

For genotyping of the *GHR exon 3* locus, a simple multiplex PCR assay was used as previously described (2). Amplification products were analysed by electrophoresis on a 1% agarose gel stained with ethidium bromide. *GHR fl* allele was detected as a single band corresponding to 935 bp and the *GHR-d3* allele corresponding to 532 bp, respectively. All samples were coded, and the operator was blinded to anthropometric data.

### Statistics

All data were assessed for normal distribution by exploring the data for skewness and kurtosis and the Shaprio–Wilks estimates. Infants were defined as SGA and large for gestational age (LGA) if birth weights were below 10th centile or above 90th centile, respectively, for gestational age. Postnatal catch-up or catch-down growth was defined as an increase or decrease in weight SDS of 0·6 over the first year of life.^13^ In the UCL FGS cohort, Student’s *t*-test was used to compare the GHR genotyped group (*n* = 774) and total cohort (*n* = 1674) mean values to ensure there was no selection bias in those samples used for genotype analysis.

Chi-square tests were used to compare frequency distributions of the *GHR* genotypes. One-way analysis of variance (anova), with the Tukey’s honest significant differences (HSD) *post hoc* test, was used to determine differences between the mean anthropometric measures in the *GHR exon 3* genotypes. Multiple stepwise linear regression was used to determine factors influencing placental weight and birth weight SDS.

A sample size of 1048 was estimated to have a power of 90% to calculate a 50 g difference in placental weight and 100 g difference in birth weight at 5% level of significance between SGA and LGA groups.

## Results

### General

Both cohorts consisted of a total of 1048 singletons with equal number of men and women. Maternal and infant cohort data shown in Table 1 confirm similarities of UCL-FGS and Moore cohort. In the UCL-FGS, there was no difference in these data between those who underwent GHR analysis (*n* = 774) and those remaining participants who did not (data not shown). All the data had normal distribution and hence log transformation was not required.

**Table 1:** Maternal and infant anthropometric and demographic data from the UCL-FGS and the Moore cohort

	UCL-FGS *n* = 774	Moore cohort *n* = 274
Maternal parameters		
Maternal height (cm)	164·6 (6·90)	165·8 (6·25)
Maternal weight (kg)	63·9 (11·9)	66·8 (11·9)
Infant parameters		
Gestation (weeks)	39·4 (1·05)	39·1 (1·00)
Placental weight (g)	699·2 (136·8)	708·8 (137·7)
Birth weight SDS	0·12 (0·98)	0·17 (0·93)
Birth length SDS	−0·12 (0·75)	–
Head circumference SDS	−0·005 (0·003)	−0·01 (0·004)

Data shown as mean with SD in parentheses.

UCL-FGS, University College London Hospital Fetal Growth Study; SDS, standard deviation scores.

### Infant GHR isoforms

The frequency distribution of the *GHR exon 3* genotype was as follows: *fl/fl* 48%, *fl/d3* 45% and *d3/d3* 7%. The genotype distribution was in Hardy–Weinberg equilibrium. Parity and cigarette smoking were distributed similarly across the *GHR genotype* (χ^2^ = 0·12, *P* = 0·94, df = 4 and χ^2^ = 0·18, *P* = 0·91, df = 4 respectively).

In the UCL-FGS, *GHR* genotype was not associated with parameters of antenatal growth (Table 2). The effect of infant’s *GHR* genotype on anthropometric measures on the entire cohort at birth is shown in Table 2. *GHR exon 3* genotype was significantly associated with placental weight (one-way anova*F* = 7·557; *P* = 0·001, df = 992) and birth weight SDS (one-way anova*F* = 3·3; *P* = 0·037, df = 1047). In infant’s placenta, weight and birth weight SDS was higher in the *GHR fl/fl* genotype group and lower in the *GHR d3/d3* genotype. *GHR* genotype was not significantly associated with birth length SDS (one-way anova*F* = 1·6; *P* = 0·2, df = 1020) or head circumference SDS (one-way anova*F* = 0·865; *P* = 0·42, df = 1021).

**Table 2:** Association of infant *GHR exon 3* genotype with antenatal growth (*n* = 774), placental weight and anthropometric measurements at birth (*n* = 1048)

	*GHR exon 3* genotype				
	
Anthropometric measurements	*GHR fl/fl* (48%)	*GHR fl/d3* (45%)	*GHR d3/d3* (7%)	df	*P* value
First trimester (12–14 weeks of gestational age)					
Femur length (cm)	1·9 (0·6)	1·8 (0·55)	1·8 (0·65)	754 (2)	0·55
Abdominal circumference (cm)	14·3 (1·4)	13·7 (2·3)	13·1 (2·0)	759 (2)	0·66
Bi parietal diameter (cm)	3·0 (0·7)	2·9 (0·7)	2·9 (0·6)	760 (2)	0·51
Second trimester (18–20 weeks of gestational age)					
Femur length (cm)	3·3 (0·3)	3·3 (0·3)	3·2 (0·2)	750 (2)	0·77
Abdominal circumference (cm)	15·7 (1·3)	15·7 (1·3)	15·6 (1·0)	759 (2)	0·83
Bi parietal diameter (cm)	4·95 (0·32)	4·95 (0·42)	4·90 (0·22)	760 (2)	0·61
Third trimester (30–34 weeks of gestational age)					
Femur length (cm)	6·2 (0·3)	6·2 (0·3)	6·1 (0·4)	700 (2)	0·46
Abdominal circumference (cm)	28·7 (1·8)	28·6 (1·7)	28·4 (2·1)	700 (2)	0·70
Bi parietal diameter (cm)	8·5 (0·43)	8·4 (0·38)	8·3 (0·50)	615 (2)	0·08
Birth					
Placental weight (g)	697 (142)	665 (131)	656 (136)	990 (2)	0·001*
Birth weight SDS	0·22 (1·0)	0·14 (0·95)	0·05 (0·90)	1045 (2)	0·037*
Birth length SDS (UCL-FGS, *n* = 774)	−0·04 (1·17)	−0·18 (1·14)	−0·21 (1·08)	1018 (2)	0·2
Head circumference SDS	0·05 (1·13)	−0·05 (1·09)	−0·04 (0·95)	1019 (2)	0·42

Data shown as mean with SD in parentheses, *P* value calculated by one-way analysis of variance (anova).

SDS, standard deviation scores; UCL-FGS, University College London Hospital Fetal Growth Study; df, degree of freedom within-group with between-group combined in parentheses.

* Significant *P* value. Antenatal growth parameters analysed in UCL-FGS *n* = 774. Placental weight and postnatal growth analysed in UCL-FGS and Moore cohort, *n* = 1048.

**Figure 1:**
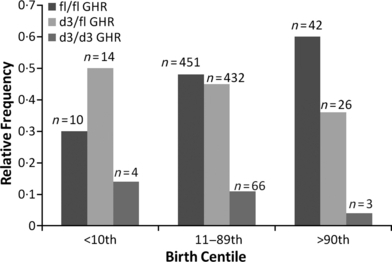
Frequency of *GHR exon 3* genotypes based on birth weight centile. Chi-square *P* value; <10th centile – 0·003, >90th centile – 0·047, df = 2.

There was a greater proportion of *d3/d3* (14%) and lower proportion of *fl/fl* (36%) in the SGA group at birth (χ^2^ = 11·2, *P* = 0·003, df = 2). In the LGA category the *fl/fl* genotype was overrepresented (60%) and *fl/d3* and *d3/d3* genotypes were underrepresented (χ^2^ = 6·1, *P* = 0·047, df = 2) (Fig. 1).

In the UCL-FGS, no association of *GHR* genotype was found with postnatal anthropometric data up to 3 years of age (data not shown). *GHR* genotype was also not associated with catch-up (χ^2^ = 0·28, *P* = 0·87, df = 2) or catch-down (χ^2^ = 0·58, *P* = 0·75, df = 2) growth in the first year of life.

In multiple regression analyses (adjusted *R*^2^ 35%), *GHR* genotype, booking weight, parity and gestational age of delivery were associated with placental weight while maternal age and cigarette smoking did not influence placental weight (Table 3). In multiple regression analyses, birth weight SDS was not influenced by *GHR* genotype (data not shown).

**Table 3:** Multiple stepwise linear regression analyses of factors influencing placental weight

Covariate	Coefficient	*t*-ratio	*P* value
*GHR* genotype	−0·13	−3·482	0·001
Booking weight	0·12	3·15	0·002
Gestational age at delivery	0·28	7·55	0·0001
Parity	0·14	3·73	0·0001

Degree of freedom for regression = 7, residuals = 664.

### Maternal GHR isoforms

Maternal *GHR* genotype did not demonstrate any significant association with placental weight or any anthropometric measures at birth (Table 4).

**Table 4:** Association of maternal *GHR exon 3* genotype with placental weight and infant anthropometric measure at birth

	*GHR exon 3* genotype			
	
	*GHR fl/fl* (49%)	*GHR fl/d3* (40%)	*GHR d3/d3* (11%)	*P* value
Placental weight (g)	691 (134)	685 (130)	705 (137)	0·2
Birth weight SDS	0·2 (1·0)	0·7 (0·85)	0·36 (0·90)	0·09
Birth length SDS (UCL-FGS)	−0·03 (0·27)	−0·18 (1·04)	−0·21 (1·08)	0·12
Head circumference SDS	−0·05 (0·13)	−0·15 (1·09)	0·12 (0·85)	0·42

Data shown as mean with SD in parentheses, *P* value calculated by one-way analysis of variance (anova).

SDS, standard deviation scores; UCL-FGS, University College London Hospital Fetal Growth Study.

## Discussion

In a cohort of white European pregnant women and their offspring, we found a significant positive association between *GHR exon 3* genotype and placental and birth weight. This genotype appears to influence intrauterine growth in late gestation as our study demonstrated no clear effect on ultrasonographic measures of antenatal growth up to the third trimester of pregnancy (30–32 week scan). Further, the effect does not seem to be maintained into postnatal life as no long-term effect on size could be determined nor on the catch-up and catch-down growth process.

Adequate placentation and placental growth are essential for foetal growth. There is a strong correlation between placental weight and foetal growth with most low birth infants having a small placenta.^14^,^15^ This implies that placental growth is linked to foetal growth. The placenta expresses GHR, which can bind the increasing concentrations of placental growth hormone (hGH-V) encoded by chorionic somatomammotropin hormone 1 (CSH1), (MIM: *150200) during pregnancy. hGH-V is secreted by syncytiotrophoblasts and its concentration progressively rises in maternal plasma from 10–12 weeks of gestation to term.^16^ hGH-V is also expressed in invasive extravillous trophoblasts suggesting that the physiological role of hGH-V, in addition to endocrine effects in the mother, might also include a direct influence on placental development via an autocrine or paracrine mechanism.^17^ hGH-V is expressed in the placenta^18^ and could potentially influence autocrine or paracrine effects of placental or foetal GH on placental growth.^10^ Circumstantial evidence for this comes from the observation that deletion of the *PGH* gene in the foetus is associated with marked foetal growth restriction and a small placenta^19^ and that the placental weight of *GHRfl/fl* group in our study was heavier than that of the *GHRd3/d3* group.

Our data would suggest that the size of the effect on birth weight is less than that upon the placenta but given the interaction of placental weight and birth size it is not easy to dissect this further. In the multiple linear regression model, placental weight is influenced by *GHR* genotype, while birth weight SDS is not suggesting that *GHR* genotype effect on birth weight could be mediated through placental weight.

Jensen *et al.*^10^ have demonstrated an influence of *GHR exon 3 deleted* genotype on foetal growth velocity in SGA infants and increased postnatal growth in both SGA and appropriate for gestational age (AGA) infants. Sorensen *et al.*^11^ have shown decrease in birth weight and birth length in *GHR d3/d3* genotype similar to our study. Unlike other studies, we could not demonstrate any effect on prenatal weight up to 32 weeks of gestational age and postnatal growth. This probably reflects the fact that our cohort represents a continuum across the birth size spectrum in the general population and does not represent polarized size class groups. In addition, the larger sample size in our study reduces the likelihood of false-positive findings.

Of note, large genome wide association studies (GWAS) have not found any influence of *GHR* locus on height or metabolic parameters.^20^ This could simply mean that *GHR* variation does not significantly influence growth or the SNP that tags *GHR exon 3* in the GWAS does not recapitulate all the genomic variability at the locus, including the *d3*, other copy number variations, or rare variants.^21^

In conclusion, we found that the *GHR exon 3* polymorphism genotype is associated with placental weight and birth weight with carriers of *GHRfl/fl* allele having a heavier placenta and higher birth weight. Placental weight could be potentially influenced by an autocrine/paracrine action of hGH-V or other products of the chorio-somatomammotropic gene on the *GHR* polymorphism present in the placenta.
